# Computational chemistry, data mining, high-throughput synthesis and screening - informatics and integration in drug discovery

**DOI:** 10.1155/S1463924601000256

**Published:** 2001

**Authors:** Charles J. Manly

**Affiliations:** Discovery Technologies Neurogen Corporation Branford CT USA

## Abstract

Drug discovery today includes considerable focus of laboratory automation and other resources on both combinatorial chemistry and high-throughput screening, and computational chemistry has been a part of pharmaceutical research for many years. The real benefit of these technologies is beyond the exploitation of each individually. Only recently have significant efforts focused on effectively integrating these and other discovery disciplines to realize their larger potential. This technical note will describe one example of these integration efforts.


Journal of Automated Methods & Management in Chemistry, Vol. 23, No. 6 (November–December 2001) pp. 191–192

### Technical note
### Computational chemistry, data mining, high-throughput synthesis and screening—informatics and integration in drug discovery†

**Charles J. Manly**
*Discovery Technologies, Neurogen Corporation, Branford, CT, USA*

*Drug discovery today includes considerable focus of laboratory automation and other resources on both combinatorial chemistry and high-throughput screening, and computational chemistry has been a part of pharmaceutical research for many years. The real benefit of these technologies is beyond the exploitation of each individually. Only recently have significant efforts focused on effectively integrating these and other discovery disciplines to realize their larger potential. This technical note will describe one example of these integration efforts.*

### Introduction

Neurogen Corp. is a pharmaceutical company focusing on central nervous system (CNS) disorders. Several years ago, it began to develop methodology, now named 'AIDD$^{sm}$' (accelerated intelligent drug discovery), with the aim of streamlining and optimizing:

- the generation of lead series;
- the exploration and characterization of lead series;
- the optimization of leads; and
- the optimization of clinical development candidates.

AIDD$^{sm}$ accomplishes this through tight integration (via intranet deployed informatics) of combinatorial chemistry, high-throughput pharmacology and computational chemistry. AIDD$^{sm}$ itself is tightly integrated with the drug-discovery effort and especially with medicinal chemistry itself (figure 1).

The focus of AIDD$^{sm}$ is on the ability to enhance greatly the drug-discovery cycle: synthesis, data generation, data analysis and modelling and prioritization of both synthesis and screening—thus completing the cycle—on thousands of compounds every 2 weeks. Additionally, this is accomplished with:

- very small staff resources (20–25 FTEs);
- the ability to synthesize 400 000 samples per year (as either mixtures or individual samples) with purification and quality assessment;
- biological data generation of 300 000 samples per month;
- a cycle time of 2 weeks;
- targeted efficiency gains through computational chemistry and data-mining of 10 × to well over 50 × over random; and
- the ability to prosecute 13–15 programmes simultaneously in the above manner.

† This technical note was initially presented at the ISLAR 2000 Conference and is reproduced here by kind permission of Zymark Corporation.

### Virtual library (figure 2)

The AIDD$^{sm}$ virtual library is managed by Neurogen's ISLANDS$^{sm}$ technology, and is a representation of all compounds that can be made from the existing reactive fragment database and synthesis protocols database. Thus, this virtual library is a very specific and dynamic set of compounds that can easily be millions or billions of molecules in size. The ISLANDS$^{sm}$ technology managing the virtual library is key to AIDD$^{sm}$ virtual screening processes as well as to workflow operations. The ISLANDS$^{sm}$ software makes it possible to define and register 50 000 compounds from the virtual library easily and quickly (10 min). After definition and registration, not only do the compounds exist electronically in databases for use in AIDD$^{sm}$, but also ISLANDS$^{sm}$ has generated all information required in the synthesis itself. The reagents required, the synthesis, reaction work-up and quality control protocols to be used by the synthesis

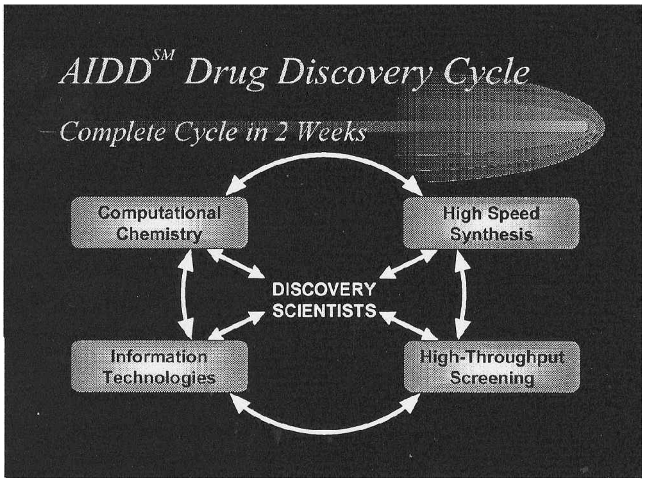

*Figure 1.*

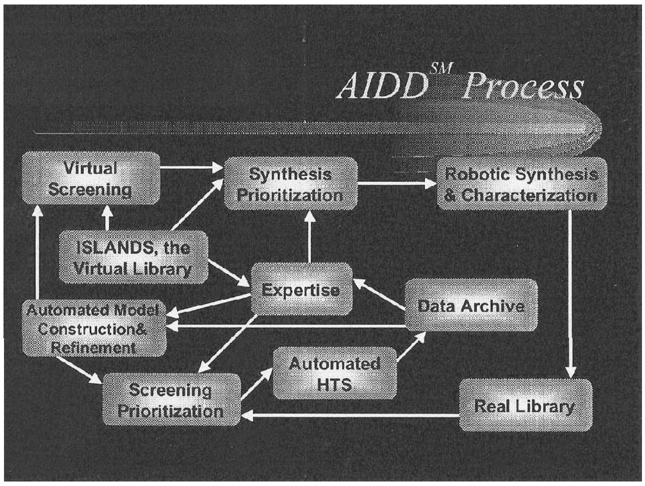

Figure 2.

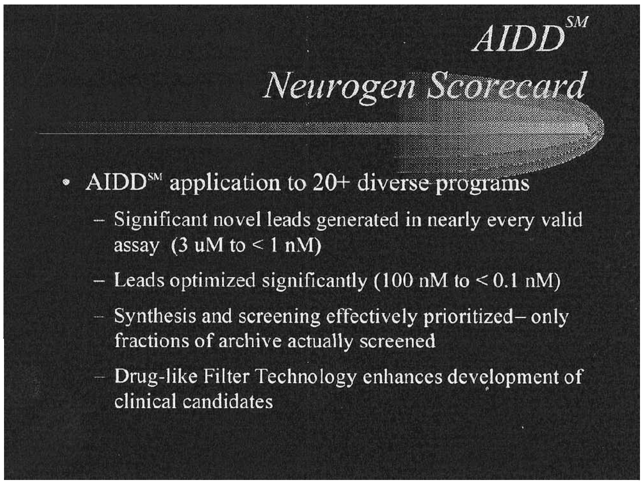

Figure 3.

robotics and all tracking information (sample number, plate number, well locations) have been automatically generated and specified with no further input from the user required.

### Virtual screening

A key concept of AIDD$^{sm}$ is the effective prioritization of both synthesis and screening resources through virtual screening. Proprietary, unattended and continuous molecular modelling and data-mining strategies termed 'on-line continuous modelling' (OLCM) provide models for virtual screening of both the virtual library and the archive of actual compounds. These models work in concert with ISLANDS$^{sm}$ for virtual screening of the virtual library.

### On-line continuous modelling

From the inception of our work on AIDD$^{sm}$, we planned to perform computational chemistry modelling with a novel portfolio approach. A portfolio of modelling strategies could be expected to provide useful models in a variety of cases when no one strategy could be expected to perform well in every situation. Compare this with a stock portfolio where the expectation is that the portfolio will increase in value with time even though this cannot be expected of any one particular stock. The AIDD$^{sm}$ portfolio of OLCM includes a variety of both chemical descriptor types and modelling methods. Fuzzy methods and machine methods have been very effective. Both articifical neural networks and recursive partitioning methodologies are also used routinely in AIDD$^{sm}$ OLCM studies.

A core principle of AIDD$^{sm}$ and OLCM is the prediction, prioritization and targeting of populations of compounds instead of individual compounds. This makes it possible routinely to achieve significant benefits by increasing the probability of activity in each 2-week cycle. Efficiency gains or targeting enhancements seen in AIDD$^{sm}$ from this approach are routinely $10\times$ to more than $50\times$ enhancement.

### Results

AIDD$^{sm}$ has been applied to over 20 diverse programmes at Neurogen. In almost every programme, the value of AIDD$^{sm}$ has resulted in novel leads that were readily optimized to significant levels of activity (figure 3).

For the last few years, Nurogen has been applying AIDD$^{sm}$ technology to the optimization of drug-like properties within projects toward the generation of development candidates. OLCM models for several of these drug-like properties provide guidance for optimization of chemical series. These efforts have resulted in more efficient optimization of candidate ADME, toxicological and PK properties such as metabolic half-life, cytochrome P450 activity and others.

### Summary

An overview of the AIDD$^{sm}$ drug discovery system at neurogen has been given. Specific examples from active project areas were presented. The importance of integration of disciplines and of pragmatism in balancing the individual components of drug discovery was stressed.

